# Crossing Over…Markov Meets Mendel

**DOI:** 10.1371/journal.pcbi.1002462

**Published:** 2012-05-17

**Authors:** Saad Mneimneh

**Affiliations:** Department of Computer Science, Hunter College of CUNY, New York, New York, United States of America; Whitehead Institute, United States of America

## Abstract

Chromosomal crossover is a biological mechanism to combine parental traits. It is perhaps the first mechanism ever taught in any introductory biology class. The formulation of crossover, and resulting recombination, came about 100 years after Mendel's famous experiments. To a great extent, this formulation is consistent with the basic genetic findings of Mendel. More importantly, it provides a mathematical insight for his two laws (and corrects them). From a mathematical perspective, and while it retains similarities, genetic recombination guarantees diversity so that we do not rapidly converge to the same being. It is this diversity that made the study of biology possible. In particular, the problem of genetic mapping and linkage—one of the first efforts towards a computational approach to biology—relies heavily on the mathematical foundation of crossover and recombination. Nevertheless, as students we often overlook the mathematics of these phenomena. Emphasizing the mathematical aspect of Mendel's laws through crossover and recombination will prepare the students to make an **early** realization that biology, in addition to being experimental, IS a computational science. This can serve as a first step towards a broader curricular transformation in teaching biological sciences. I will show that a simple and modern treatment of Mendel's laws using a Markov chain will make this step possible, and it will only require basic college-level probability and calculus. My personal teaching experience confirms that students WANT to know Markov chains because they hear about them from bioinformaticists all the time. This entire exposition is based on three homework problems that I designed for a course in computational biology. A typical reader is, therefore, an instructional staff member or a student in a computational field (e.g., computer science, mathematics, statistics, computational biology, bioinformatics). However, other students may easily follow by omitting the mathematically more elaborate parts. I kept those as separate sections in the exposition.

## Introduction

### Mendel and High School Biology

**Figure 1:**
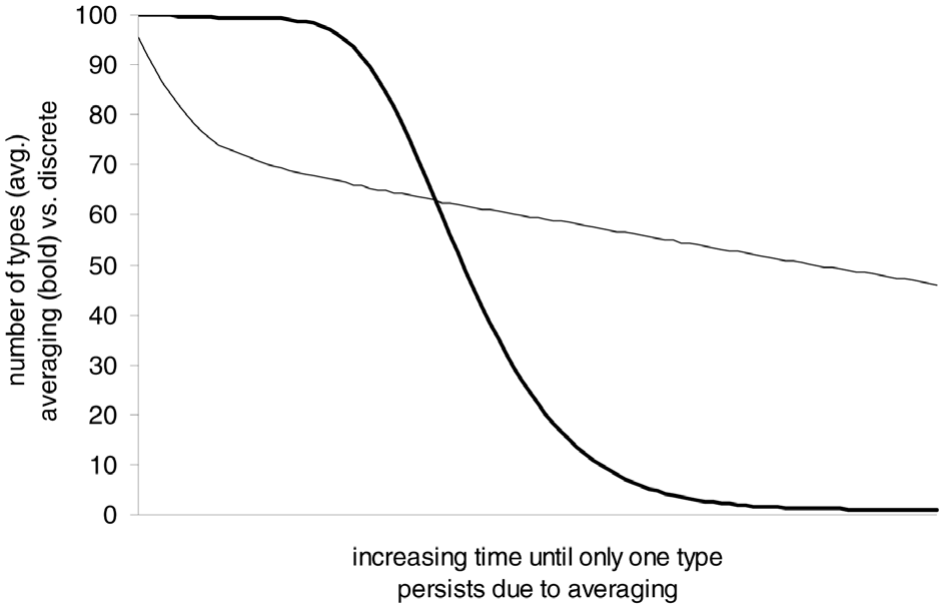
Fast convergence of inheritance by averaging.

Sexually reproducing organisms generally combine heritable traits from two parents. The biological process that combines those traits is called meiosis. While mutations could occur during meiosis, most of the variation arises from the combinations of parental traits. How do these parental traits combine? The dominant theory was that some sort of blending or averaging took place. However, such a mode of inheritance would result in an average of all ancestors after only a modest number of generations (imagine repeatedly mixing colors). Instead, by performing experiments on plants, Mendel pointed out the existence of discrete elements that combine but do not mix. Figure 1 shows the simulated number of types of individuals as a function of time. Averaging, with traits taking real values in 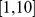, is used on one population, and the model described in the section “A Simple Model”, with elements (later called alleles) taking discrete values in 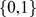, is used on another. Mutations are ignored. In both cases, a population size of 100 is kept constant for the entire duration of the simulation (100 time steps). The simulation is repeated 1,000 times to obtain an average for each time step.

Mendel formulated the concept of a *gene* (unit of inheritance), and hypothesized that inheritance is governed by the following two laws of *gene*tics:

1. **Segregation**: Each sexually reproducing organism has two *alleles* (copies) for each gene, one inherited from each parent; and in turn will contribute, **with equal probability** (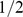), only one of these two alleles.
2. **Independent assortment**: Alleles of different genes are inherited **independently** (later deemed not so accurate).

The state of a gene, the *genotype*, is determined by the two alleles. The resulting trait, the *phenotype*, is then a function of this state. When the alleles are the same, the gene, or equivalently the genotype, is *homozygous*; otherwise, it is *heterozygous*. For example, if an allele can be either 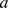 or 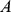, then the possible genotypes are 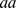, 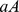, 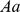, and 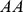. Table 1 shows the possible segregations of parental genotypes when at least one of them is heterozygous.

**Table 1:** Genotypes.

	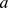	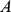			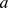	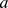			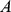	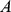
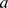	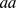	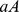		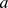	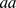	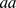		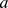	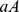	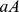
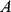	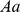	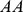		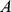	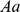	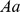		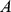	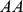	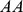

In a dominant/recessive mode where 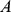 is dominant, the corresponding phenotype is obtained as a function of the genotype as shown in Table 2, leading to a 3∶1 ratio, a 1∶1 ratio, and a 1∶0 ratio of dominant to recessive phenotypes, respectively.

**Table 2:** Phenotypes.

	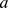	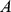			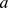	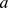			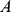	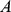
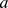	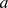	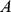		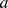	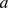	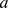		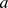	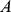	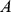
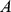	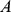	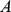		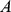	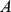	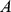		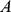	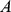	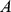

Students often overlook that these ratios are not simply based on counting the entries, but the result of the segregation law: each allele is contributed with equal probability, i.e., 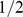, resulting in a probability of 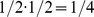 for each entry in the tables. Table 3 shows another example involving two heterozygous dominant/recessive genotypes that lead to a 9∶3∶3∶1 ratio of phenotypes. In addition to the segregation law, students should be reminded that this ratio assumes that the law of independent assortment holds: alleles of different genes are inherited independently, resulting in a probability of 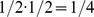 for each assortment (refer to the next section for a mathematical definition of independence), thus a probability of 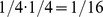 for each entry in the table.

**Table 3:** Phenotypes for two heterozygous genotypes.

			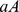	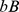	
		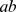	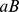	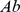	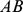
	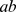	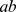	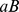	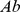	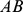
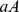	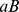	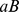	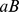	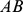	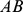
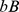	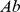	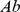	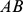	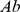	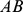
	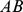	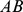	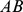	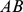	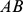

### Chromosome, Crossover, and Recombination

About 100 years later, it was established that the physical structure underlying Mendel's laws is the chromosome (for simplicity, a long molecule of DNA). This discovery matched Mendel's experiments really well: In diploid organisms like us chromosomes come in pairs (thus the name diploid), one from each parent! With few exceptions, each chromosome of the pair has copies of the same genes (special stretches of DNA) arranged in the same order: the alleles! In an attempt to explain experimental results and confirm Mendel's laws, chromosomal *crossover* was formulated and described by Thomas Morgan (coincidentally, his student John Northrop was a teacher of botany at Hunter College, the author's institution), but demonstrated only about 20 years later. Crossover is a mechanism that occurs at the early stages of the meiotic prophase, and combines the two chromosomes of the pair into one, a process called *genetic recombination*. During this process, the chromosome of the pair that is the source of the allele alternates every so often. Exactly when the switch—the crossover—happens is almost arbitrary.

**Figure 2:**
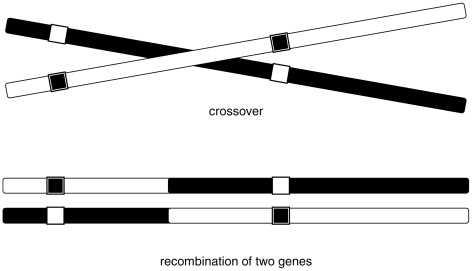
One chromosomal crossover and a genetic recombination.

When two alleles come from different chromosomes of the pair, their corresponding genes are said to recombine (can you identify the recombinations in Table 3?). Figure 2 illustrates a genetic recombination with one crossover.

### A Slight Discrepancy and Genetic Linkage

Mendel's laws (segregation and independent assortment) dictate that genetic recombination occurs with a probability of 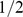. Let's re-examine why this holds true. Let 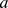 and 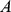 be the two alleles of gene 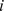 on the two chromosomes. Similarly, let 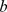 and 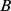 represent the same for gene 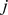, respectively. Chromosomal crossover will result in recombination of gene 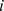 and gene 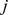 if one of the two assortments 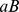 and 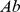 occurs. Since each allele is contributed with equal probability (segregation), both 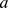 and 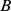 are contributed with probability 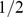. Since alleles of different genes are inherited independently (independent assortment), the assortment 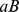 occurs with probability 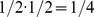 (refer to the next section for a mathematical definition of independence). The same analysis applies for the assortment 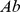, leading to an overall recombination probability of 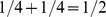.

However, it has been observed that some pairs of genes show a correlation in their alleles, e.g., their probability of recombination is less than 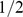. In this case, there is a *linkage* between the genes. How can we now incorporate this notion into the mathematics of Mendel's laws, which so far have relied on the fact that genes are not correlated (assorted independently)? Fortunately, a simple probabilistic model based on Figure 2 (1 crossover) will capture the effect of linkage, and as a result, alleles that are near each other on a chromosome will tend to be inherited together. The inaccuracy of Mendel's law of independent assortment lies therein. Nevertheless, one should still expect that genes which are far from each other on a chromosome (or on different chromosomes altogether) will assort independently, as Mendel once observed. It will require a better probabilistic model to reflect those two contradictory behaviors (genetic linkage and independence); the later introduction of the Markov chain will take care of this. But first, I will present a simple probabilistic model for genetic linkage. And before doing so, let's review some basic mathematics.

## What Do We Need to Know?

### Probability

Let 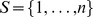. A subset of 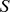, 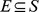, is considered as an event (but not all events are subsets of 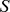). Given a variable 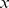, define the following probabilities of events:
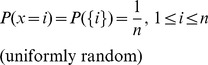


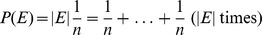
where 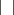 denotes the size of a set. So 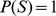. The negation of an event will always satisfy:
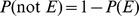
Given two events 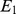 and 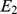, 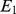 and 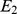 are exclusive (cannot occur together) if and only if
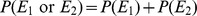
Given two events 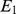 and 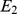, 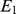 and 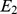 are independent if and only if
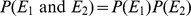
For instance, if 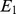 is an event of probability 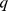 and 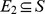, then 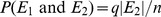. In general, however, 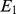 and 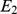 may not be independent. So we define the probability of 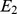 conditional on 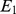, i.e., the probability of 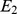 given that 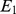 occurs.
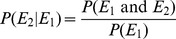
For instance, let 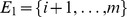 and 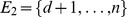 with 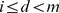. Note that 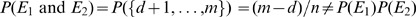. Then,
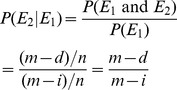



### Matrix Multiplication

I will assume some familiarity with matrices. If, however, this notion is unfamiliar, the parts of the exposition that use matrices may be skipped. Only 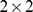 matrices will be considered in this exposition. The multiplication of 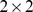 matrices is defined below.
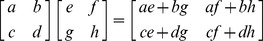


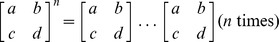



### Geometric Series

One of the series that is almost invariably covered in basic calculus is the geometric series.
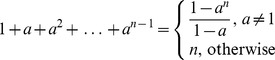



### Exponential Limit

This is one of the basic expressions covered when studying limits.
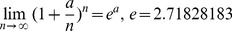
Therefore, 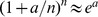 for large 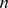.

### Logarithm

Here's the definition of natural logarithm and some of its properties:
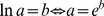


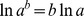


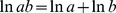



### Harmonic Series

Another famous encounter is the harmonic series and its approximation.
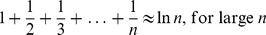



### Derivatives

A function 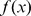 reaches a local maximum or minimum when its derivative 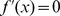. Here are some examples of derivatives:
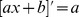


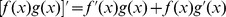


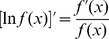



## A Simple Model

Motivated by Figure 2, a uniform 1-crossover model can be constructed as follows: Consider a chromosome with 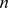 genes, i.e., 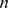 alleles on each chromosome of the pair. A crossover 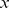 is equal to 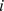 if it separates gene 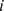 and gene 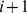, where gene 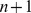 is hypothetical when 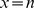, i.e., no crossover. Assume that 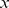 is uniform in 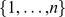 (thus the name of the model).

### Linkage

Based on the above setting, 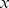 takes any value in 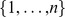 with probability 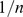. Two genes at a distance 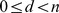, say 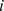 and 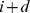, will recombine if 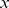 is in 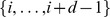, i.e., with probability 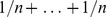 (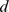 times),
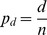
This confirms that genes within a close distance (small 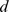) on the chromosome are less likely to be subject to recombination (genetic linkage). Genes that are far apart (large 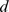) have a high probability (up to 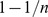) of recombination, but are they independent (see “What Is Wrong” section)?

### Segregation

To find the probability that a given allele of gene 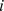 is inherited, let 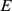 with probability 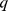 be the event that the recombination process starts on the given chromosome of the pair. This event and that genes 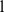 and 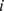 recombine (an event of probability 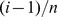) are independent. The probability of inheriting the given allele is:
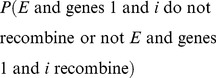


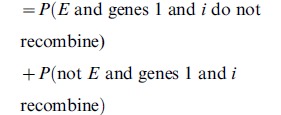
The addition is justified by the exclusivity of the events: a given allele is inherited when the process starts on the given chromosome and genes 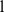 and 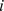 do not recombine, or when the process starts on the other chromosome and genes 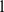 and 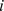 recombine. Due to the independence of 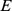 and recombination, the above becomes:
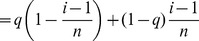
A reasonable assumption is that 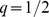 and, in this case, the above evaluates to 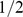 for every 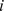, as predicted by the segregation law.

## Genetic Mapping

Genetic mapping is the problem of placing the genes along the chromosome in their correct relative order. The bad news: It is hard! The good news: Genetic linkage can be used to infer genetic mapping. Though obsolete (it has been done), genetic mapping can be considered to be the first effort towards a computational approach to biology. How does it work?

In the uniform 1-crossover model, genetic linkage tells us that the probability of recombination of two genes is proportional to the distance between these genes. Now consider the genotyping depicted in Table 4 where frequency of recombination can be used as a measure of distance. In a way analogous to Table 4, analyzing the frequency of different **pairs** of the phenotypes 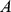, 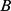, and 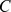 might reveal, for instance, that 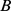 and 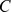 recombine more often than 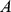 and 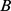; therefore, we infer that 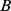 is closer to 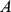 than 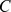. Such arguments help us to derive the gene order on the chromosome (relative order, not exact distances). While it may be hard to set up the experiment and obtain many offsprings to estimate probabilities, such arguments were definitely behind the construction of the early genetic maps, e.g., the first map of the human genome (all the chromosomes) in 1987.

**Table 4:** Frequency and distance.

			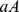	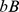	
		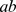	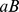	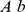	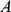 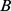
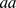 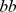	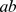	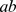	*aB*	*Ab*	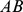

The frequency of observing *aB* and *Ab* determines the probability of recombination of the two genes, thus a measure to reflect their distance.

## What Is Wrong?

The reader may choose to skip this section to the next. The uniform 1-crossover model is very insightful in explaining Mendel's law of segregation with independent assortment corrected to reflect genetic linkage. However, it suffers from a few deficiencies.

### Linkage: OK But…

Nothing is seriously wrong about this aspect. By assigning lower probabilities of recombination for smaller distances, the distance between two genes justifies their linkage when they do not assort independently. However, the actual probability of recombination may not necessarily be **proportional** to distance or have a dependence on the chromosome length, as in 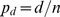 (but more on this in the Markov section).

### Segregation: Too Sensitive

The probability of inheriting a given allele is contingent on the probability that the recombination process starts on the given chromosome of the pair, previously called 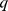. If 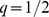, the probability of inheriting a given allele is 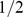, as it should be by the segregation law. While this is a biologically reasonable assumption on 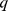, the segregation law stands very sensitive to this particular choice. A slight deviation from 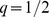 could result in a similar deviation in the probability of inheriting the given allele. Let 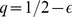, then this probability for gene 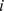 is (from the “Segregation” section):
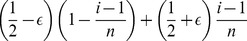
When 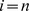, i.e., 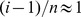, this is approximately 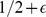. If the starting of the recombination process favors one chromosome, 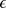 can be large, say close to 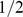 (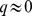). The above probability becomes arbitrarily close to 1. This means that the given allele will be inherited almost always.

### Independent Assortment: Breaks

Despite genetic linkage, one should still expect that genes which are far from each other on the chromosome will assort independently. Because each chromosome can be treated separately, this independence is certainly true for genes that are on different chromosomes altogether. But on the same chromosome, the probability of recombination 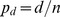 implies, for instance, that recombination of gene 1 and gene 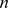 occurs with a probability of 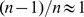 for large values of 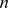. Therefore, gene 1 and gene 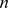 are highly correlated, and thus dependent (they will almost always recombine).

In retrospect, two genes 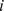 and 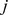 recombine when the alleles of the two genes are inherited from different chromosomes. Since the probability of inheriting a given allele is 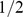 when the segregation law holds, independence then dictates that the probability of recombination of gene 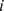 and gene 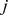 must be equal to 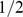. To see this, let 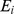 and 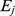 represent the events of inheriting a given allele for gene 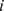 and gene 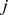, respectively, then:
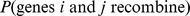


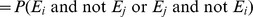


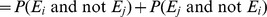


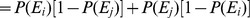
where addition is justified by exclusivity of events, and the last equality follows from that gene 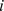 and gene 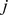 are independent. When the segregation law holds, 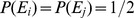 and the above expression evaluates to 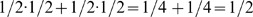. Assuming 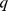 in the previous section is 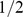, genes are independent if and only if 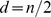. Therefore, the law of independent assortment fails when genes are on the same chromosome.

Now, why do we insist that the model must satisfy, among other properties, the law of independent assortment? Well, first because it is a correct law for distant genes. And second, since the probability of recombination increases with distance due to genetic linkage, the law of independent assortment tells us that **the probability of recombination increases up to** 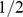 **, but cannot exceed** 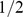 **** (this statement excludes *hotspots*, which are regions on the chromosome that experience a high probability of recombination even at small distances). It is important for students to make this realization, which will come in handy when solving genetic mapping problems, as illustrated in the section “A Computational Example of Genetic Mapping”.

### Generalization: Not Easy

One might consider extending the uniform 1-crossover model as an attempt of generalization to mimic the actual biological process. However, I will show that extending this model in the most natural way (mathematically, that is) will break the linkage property. For this purpose, consider a uniform 2-crossover model. Let 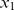 be the first crossover which is uniform in 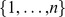 (as before), and 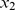 be the second crossover which, conditional on 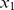, is uniform in 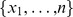. Therefore, 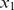 and 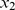 are **not independent**, for 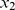 cannot precede 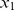. The choice of 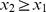 simplifies the math, but making 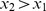 does not change the results.

Now, why even bother to show that this model, which is more difficult to analyze than its predecessor, does not work? Well, my experience in teaching has been the following: While it is important to show students what works, it is equally important to show them what does not work.

**Figure 3:**
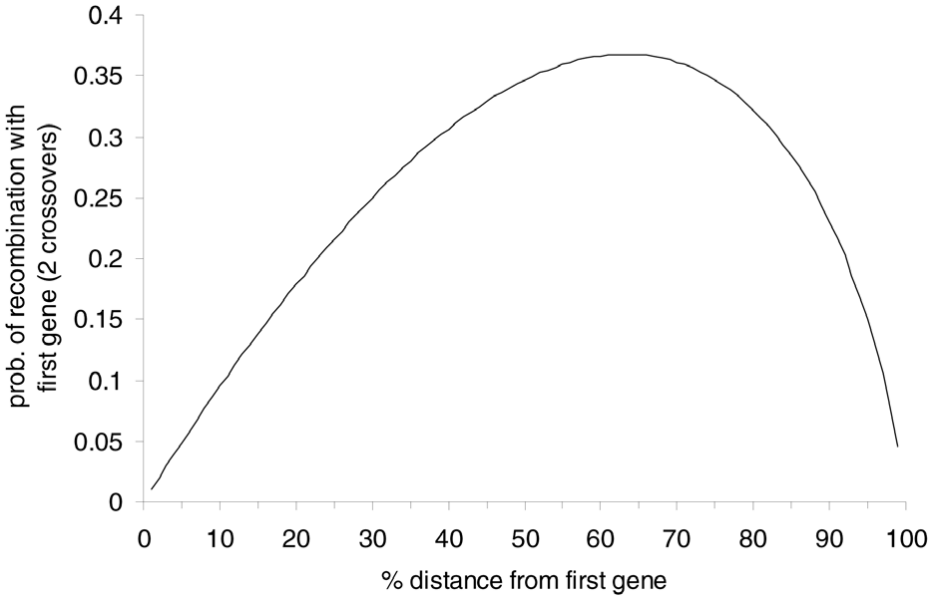
The uniform 2-crossover model. Probability of recombination of the first gene and a gene at a distance given as a percentage of the chromosome length. A maximum probability of 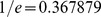 occurs at 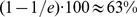.

With this in mind, all we need is a counter example, so consider gene 1 and gene 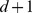 (these two genes are at a distance 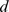 from each other). The probability of a recombination of gene 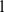 and gene 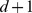 is:
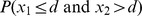
Using conditional probability and the harmonic series approximation, the “Uniform 2-crossover Model” section shows that when 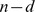 is large, this probability is approximately
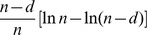
We can rewrite the above as:
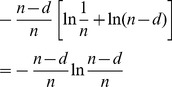
This is not an increasing function of 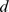. In fact, consider 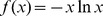. This function has a maximum of 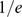 when 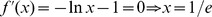. Therefore, we have the highest probability of recombination when 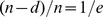, i.e., 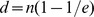. Note that in this case 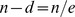, which is large (as required above) when 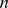 is large. This means that gene 1 is most likely to recombine with a gene located at a distance approximately 63% of the chromosome length (see Figure 3). While this is an interesting result, it stands as a pure mathematical endeavor with no biological basis.

## A Better Model: When Markov Meets Mendel

While the uniform 1-crossover model captures the essentials of segregation and linkage, it is lacking in some important aspects. First, the probability that a given allele is inherited (should be 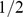) depends on an implicit parameter of the model (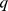 in the “Segregation” section must be 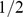). Second, genes exhibit the linkage property but they are almost never independent, as this would require a probability of recombination equal to 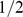 (see “A Slight Discrepancy and Genetic Linkage” section). From the “Linkage” section, this probability is expressed as 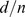, implying that only genes at a distance equal to half the chromosome length are independent. Moreover, the probability of recombination depends on the chromosome length and, therefore, two chromosomes that are locally similar but have different lengths exhibit different local recombination behavior. This is not biologically justifiable. Finally, a generalization (with uniformity maintained) to mimic the real biological process with multiple crossovers is not conceivable.

A better mathematical model is needed to rectify the above deficiencies. In principle, the model should satisfy the following three laws with multiple crossovers:

1. **Segregation**: The probability that a given allele of the gene is inherited is 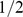.
2. **Linkage** (missed by Mendel): The probability of recombination of two genes is an increasing function of the distance between them, so it is higher for distant genes. Nevertheless, it should not depend on the chromosome length.
3. **Independent assortment**: This is impossible due to linkage where distance is a determining factor in the recombination. The alternative is to require genes to be asymptotically independent. As a result, the probability of recombination must approach 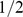 when the distance between the two genes becomes large.

Being a computer scientist by training and not a biologist, when I first suggested to my students a model based on a Markov chain, I called it the *jumping model of recombination*. I also expressed to them my concern that it may not be *real*, but as it turned out, it made perfect sense. To be loyal to my first terminology, I will call it here the jumping model.

### The Jumping Model

**Figure 4:**
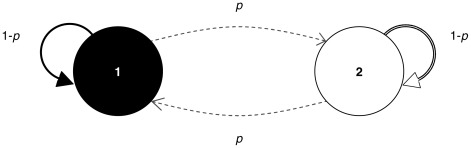
A simple Markov chain. Arguably the simplest Markov chain with two states, where each state represents one chromosome of the pair. Transitions between the two states (chromosomal crossovers) occur with probability 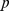.

The jumping model is based on a Markov chain. A Markov chain consists of a set of states with probabilities of transition between them (thus the jumping term). For computer scientists, this is often illustrated as a directed weighted graph with vertices representing the states and directed edges representing the transitions between states. The weight of an edge is the probability of the corresponding transition. This is shown in Figure 4 for a Markov chain with two states. Operationally, one would start at a given state and follow transitions in discrete time steps as indicated by their probabilities, thus changing state from one step to another. Let 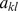 be the probability of transition from state 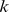 to state 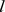, and 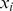 be the state at time step 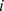. Figure 4 shows a transition probability 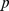 between the two states (and 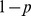 to the same state, because the transition probabilities of a given state must sum up to 1). A generalized notion of a transition is captured by a conditional probability with the following property:

**Markov property**: For 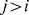,
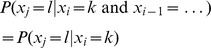
When 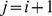, this probability is the transition probability 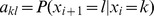. In the event 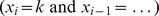 only 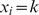 is relevant. In other words, the probability of a state at a given time depends only on the most recently known state.

**Figure 5:**
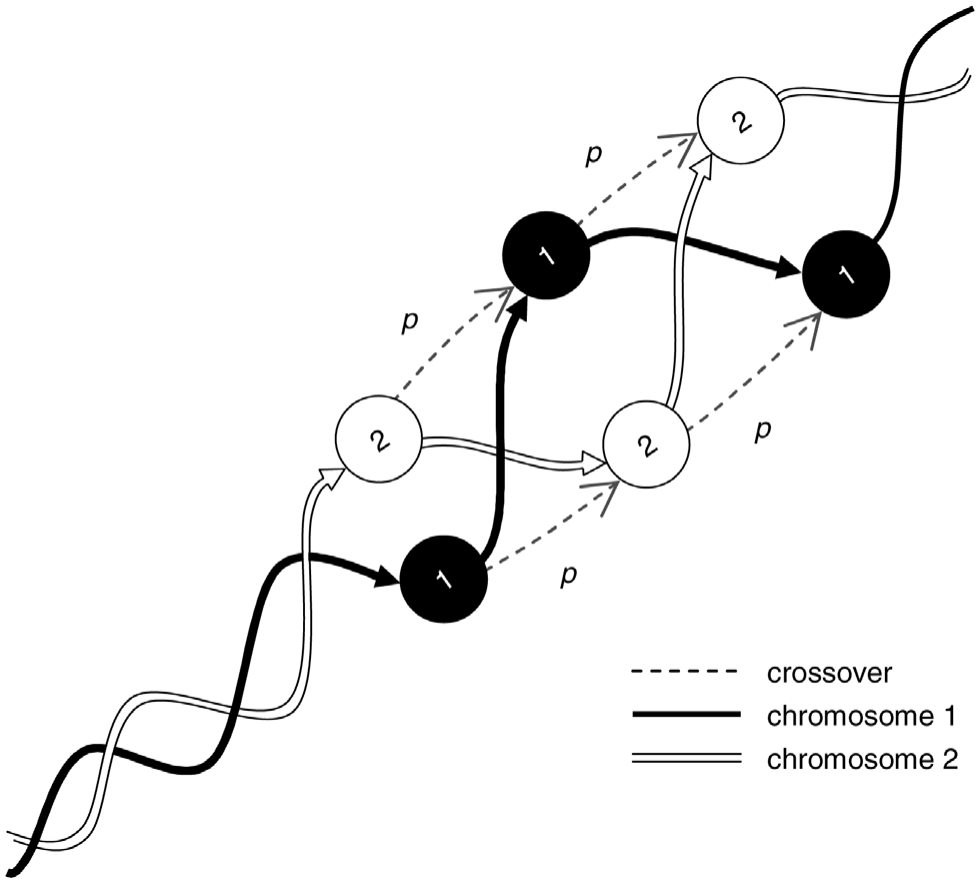
Crossover and recombination as a Markov chain. Dashed lines represent transitions (crossovers) with probability 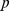, and solid lines (black and white) represent transitions (on the same chromosome) with probability 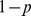.

What is the biological significance of the Markov chain in Figure 4? Each state represents a chromosome of the pair, and time in the Markov chain corresponds to genes on the chromosome. A transition between states in one time step signifies a crossover, and the probability of such a crossover is 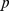. Therefore, 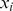 represents a crossover when 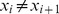. One could then inquire about the probability of being in a given state at a given time. The event of being in a given state at time 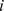 parallels the event that the corresponding chromosome is the source of the allele for gene 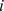. This is illustrated in Figure 5 by conceptually duplicating the chain for each gene to reflect the change of state over time.

A useful representation of a Markov chain is by a matrix 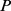 where 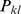 (the term in the 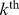 row and 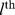 column of 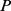) is the probability of transition from state 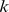 to state 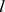; therefore, every row in 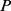 must add up to 1. If we call the states in Figure 4 state 1 and state 2, then our Markov chain can be expressed as:
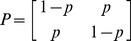
In this matrix, 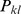 can be interpreted as 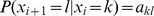. Why is this matrix representation useful? Let's multiply 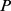 by itself:
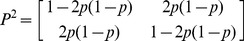
Note for instance that 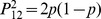 is equal to 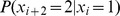, because to transition from 1 to 2 in two time steps we can transition from 1 to 1 to 2 with probability 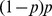 or from 1 to 2 to 2 with probability 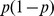. As it turns out, 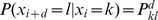. The proof of this fact is in the “Markov Transitional Probabilities” section and uses conditional probability and the Markov property. Thus, every row in 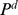 must also add up to 1.

Because 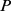 is a symmetric matrix 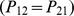, a final note is that all powers of 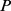 are symmetric matrices. Therefore, 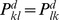, which now implies that every column in 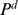 must also add up to 1. We can finally establish that the probability of recombination is
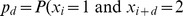


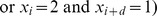


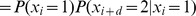


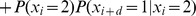


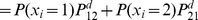


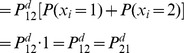



### Segregation and Independent Assortment

**Figure 6:**
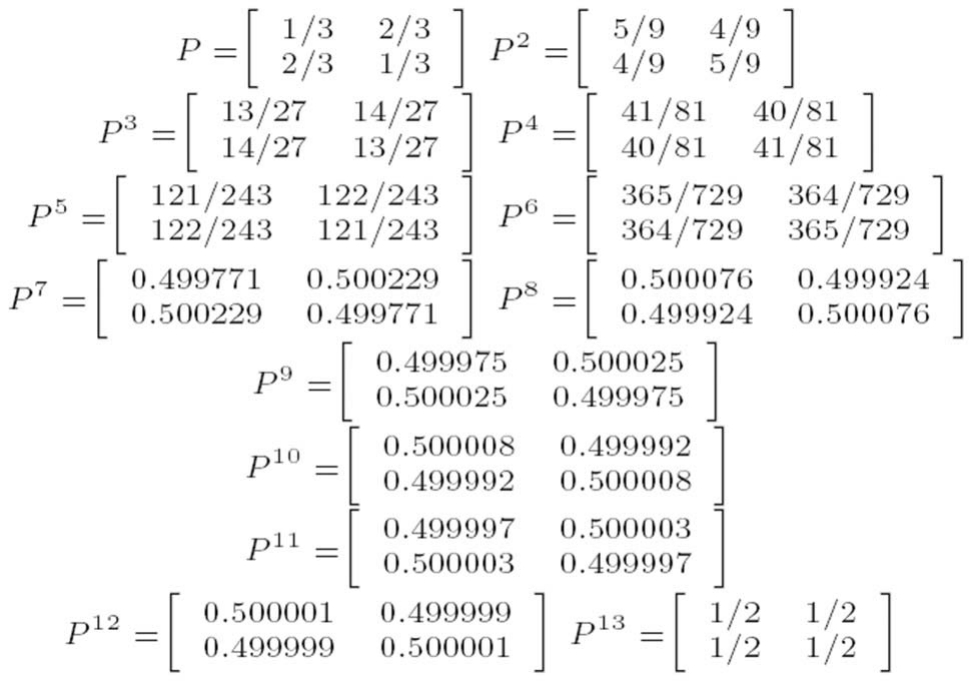
Convergence to steady state probabilities. Computation is performed with a rounding error 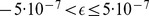.

Following the logic of previous sections, the probability that a given allele of gene 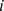 is inherited is:
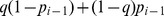
Again, if 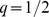 the above probability is 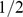, which makes the jumping model subject to the same sensitivity to 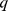 as the uniform 1-crossover model. However, this can now be alleviated. The theory of Markov chains tell us that 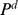 will converge for large values of 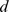 and all rows of 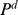 become identical. Therefore, the rows will define a *steady state* probability for each state. In other words, the effect of 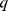 will be washed out. This theory will not be presented here, but Figure 6 shows a few powers of a given matrix 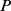.

Because 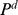 is symmetric in our case,
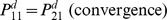


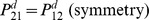


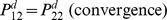
Since rows and columns of 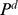 must both add up to 1, 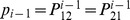 converges to 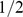 for large enough 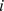. By exchanging the roles of 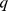 and 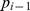 in the top expression, we also get 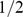, maintaining the segregation law for large enough distances when 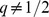.

In addition, since both 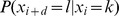 and 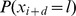 approach 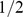, we have that 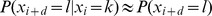 for large 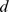. This makes 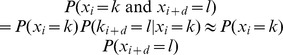 when 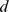 is large. Therefore, genes 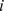 and 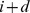 are asymptotically independent, confirming the law of independent assortment for large enough distances.

### Linkage (and Hotspots!)

The previous sections show that 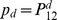 and that 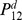 converges to 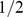 for large values of 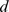, thus establishing the laws of segregation and independent assortment. However, we wish to determine 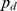 for every value of 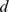. This will re-establish the above results. This time, however, and instead of using the theory of matrices (e.g., eigen decomposition) to study how 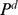 evolves, I will revert to elementary mathematics. Two genes at a distance 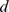 from each other will recombine if and only if their chromosome experiences an odd number of crossovers along that distance. This is equivalent to the event of making an odd number of transitions between the two states of the Markov chain during 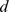 time steps. Let 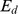 be this event (thus 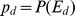). It is not hard to see that
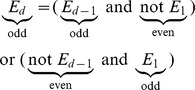
Observe that 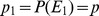. Therefore, we can write:
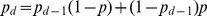
The Markov property is essential to justify the multiplication by 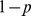 and 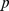 in the above equation because it makes 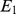 independent of the history 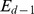. Technically, 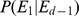 does depend on the state at time step 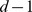, but given the symmetry in our Markov chain, it is always 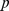. By rearranging and taking care of the special case when 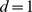 we get:
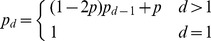
It is easy to verify that the solution
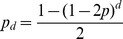
satisfies the above recurrence with a base case 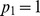 (following the pattern of the recurrence, we can retrieve the above expression if we replace 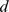 by 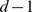, multiply by 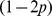, and add 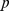).

While it is easy to verify the solution, obtaining it should not remain a wild guess. By working out a few iterations for 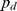, the “Recurrence for 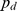
” section shows how to derive the solution using a geometric series.

**Figure 7:**
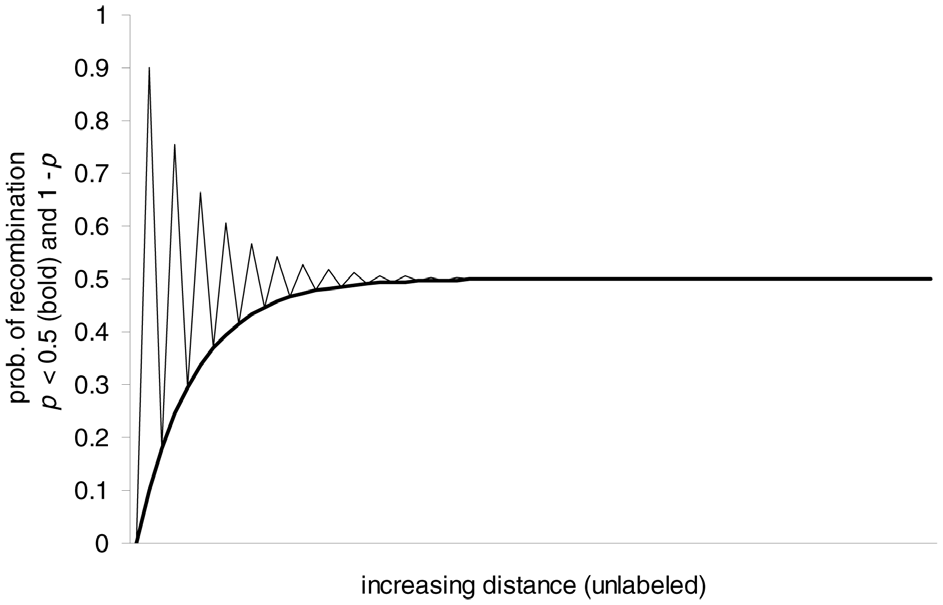
The jumping model, two modes of recombination, for 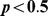 and 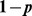.

The mathematically savvy could verify that 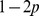 is an eigenvalue of 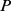, and that the same expression could have been obtained using a technique called eigen decomposition. This expression for 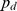 reveals interesting properties (all can be verified from Figure 7):

- When 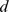 is large (and 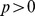), 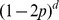 goes to zero, causing 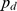 to converge to 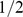. This convergence was discussed in the previous section, and should not be surprising by now.
- When 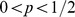 (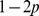 is positive), 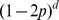 is greater than zero and less than one, causing 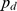 to increase with 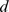 (linkage). This increase, however, is not linear as in the uniform 1-crossover model; therefore, it is biologically more realistic.
- When 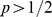 (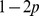 is negative), the sign of 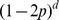 alternates, causing 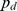 to alternate between a typical value for 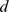 and high (hotspots, first time captured).

The jumping model captures the essential biology of crossover and recombination through the laws of segregation, linkage, and independent assortment. In addition, it reveals the non-typical high recombination probabilities of hotspots. Hotspots are regions on the chromosome that experience a high probability of recombination even at small distances. Therefore, depending on the parameter 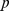, the jumping model embodies two modes of chromosomal recombination.

While a hotspot does not present a difficult concept, it is usually misinterpreted by students as a *region with high probability of recombination*. This is true if the region is too small (a peak in Figure 7), which is biologically typical of hotspots. However, if the region is large enough, there can be a high probability of recombination only if there is a corresponding low probability, as seen by the alternating pattern in Figure 7. What is interesting about the jumping model (which may not be true biologically) is that this low probability is the typical one for the given distance when 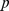 is replaced with 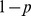. This is also confirmed by the expression we derived for 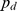, because when 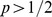 and 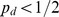, 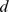 is even and, therefore, 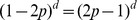:
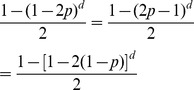
The alternation itself should be intuitive because a high probability of recombination at a small distance must be driven by a high probability of crossover, which in turn means a high probability of crossing over back to the same chromosome. The jumping model captures this fact through the parameter 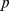 with a threshold of 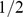 as a high probability of crossover.

### Back to the Days of Morgan

Morgan established that the probability of recombination as a function of distance is the following:
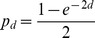
which does not account for hotspots. In addition, the notion of distance in the above expression is not the same as ours. To see this, assume that 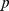 is close to zero in the jumping model (no hotspots) and, therefore, 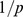 is large. Using the exponential limit,
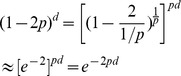
By making 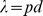, and replacing 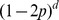 with 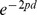 in the expression obtained for 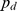, we get
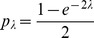
which has the same form as Morgan's expression. So what is 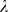
?
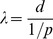
where 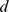 is the distance and 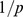 is the average distance until the next crossover (because a crossover occurs with probability 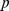). So 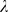 is the average number of crossovers between the two genes, and this is how Morgan defined his distance.

## Why This Way?

I could have simply argued that the probability of recombination 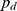 is 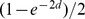, and that this is consistent with the laws of inheritance. Therefore, I will list what I believe are important aspects of this exposition.

- There is a rapid prototyping with a simple uniform 1-crossover model that reflects the essential biological properties of crossover and recombination (though not perfectly). This allows the student to quickly make a connection between the biology and the mathematics.
- There is no need for advanced calculus or probability (e.g., no mention of Poisson processes or probability distributions other than uniform).
- To achieve a better understanding of the biological properties, the exposition proceeds by pointing out the deficiencies of the simple model.
- The simple model itself is a useful tool that is actually used for simulation, e.g., genetic algorithms.
- Having a model (whether mathematical or not) provides some operational sense, so the biology is made more concrete.
- Moving progressively through the models illustrates what it takes to make attempts, including wrong ones, in the modeling of biological systems.
- Multiple models reinforce the ideas by exposing them in different settings.
- Markov chains are useful as a tool for modern biological sciences and, therefore, introducing them in this context gives the student an early preparation.
- The jumping model captures two modes of recombination, normal and hotspots, and puts them in their biological context by means of the parameter 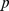.
- The jumping model also provides the insight that the probability of crossover must be less than 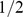 to observe the typical behavior of recombination (linkage), and hence giving the correct impression that 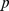 is rather small.
- The alternating behavior of the jumping model corrects one major misunderstanding of hotspots.
- Morgan's first result can be derived as a special case.
- The jumping model can be described (not necessarily analyzed) very easily and satisfies all the required biological properties of crossover and recombination. Therefore, a student can effectively retain and communicate the recombination process.

## A Computational Example of Genetic Mapping

Consider the hypothetical family in Table 5 where alleles take values in 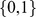 (inspired by a homework assigned by Bonnie Berger at MIT).

**Table 5:** A hypothetical family and three genes 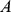, 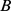, and 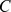 shown with their alleles.

	Genes		
	*A*	*B*	*C*
Father	0,1	0,1	0,1
Mother	0,1	0,1	0,1
Offspring 1	0,0	1,0	0,1
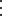	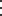	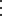	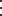
Offspring 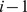	0,0	1,0	0,1
Offspring 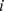	0,0	0,1	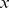 ,0
Offspring 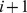	0,0	1,1	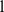 ,1
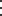	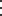	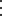	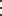
Offspring 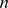	0,0	1,1	1,1

For simplicity of illustration, the chromosome of the pair with allele 0 inherited for gene 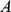 (both parental and maternal) is chosen for the offsprings, so this is not to be interpreted as if allele 0 is always inherited for gene 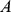. Offsprings 1 to 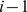 are identical, and similarly, offsprings 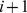 to 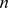 are identical. Allele 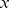 is either 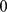 or 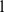, and 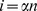 for some 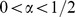.

To map the genes (genetic mapping), we count the number of recombinations, both paternal and maternal, for each pair of genes, 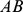, 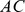, and 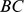. Then we estimate the probabilities of recombination and relate them to distances.

There are 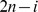 recombinations of 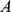 and 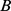, 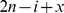 recombinations of 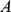 and 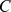, and 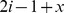 recombinations of 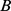 and 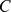. Therefore, 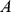 and 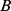 recombine with probability 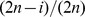, 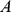 and 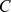 with probability 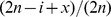, and 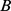 and 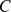 with probability 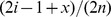. Let's denote these probabilities by 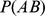, 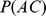, and 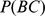, respectively. If 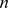 is large enough, 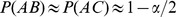 and 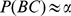.

### First Attempt

Since 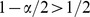 (for 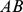 and 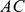), and it is not generally assumed that genes represent hotspots, we might suspect that our knowledge of the alleles of gene 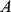 is wrong. It is more plausible that the alleles of gene 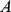 are 1,0 for the father and mother, as shown in Table 6.

**Table 6:** The same hypothetical family after the alleles of gene A have been switched.

	Genes		
	*A*	*B*	*C*
Father	**1**, **0**	0,1	0,1
Mother	**1**, **0**	0,1	0,1
Offspring 1	0,0	1,0	0,1
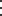	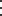	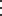	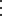
Offspring 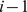	0,0	1,0	0,1
Offspring 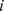	0,0	0,1	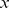 ,0
Offspring 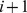	0,0	1,1	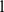 ,1
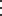	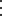	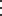	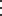
Offspring 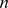	0,0	1,1	1,1

This will make 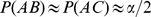 and will keep 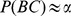. Since the probability of recombination of distant genes is higher, the order of genes is 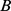, 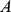, 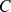 or 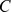, 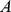, 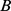.

This solution puts 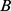 and 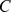 at equal distances from 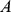 and, therefore, makes the distance from 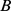 to 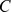 twice the distance from 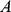 to 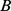 (and that from 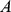 to 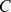). However, doubling the distance should not double the probability of recombination unless the probability is a linear function of distance like in the uniform 1-crossover model. We may adopt this model here if we know in advance that only one crossover occurs; this conditioning makes the crossover uniform even when the underlying model is the jumping one (because of the symmetry in the Markov chain). For this argument to work we will also need 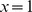; otherwise, we observe a double crossover for Offspring 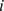 in Table 6.

### Second Attempt

If we believe that our knowledge of the alleles in Table 5 is correct, then the genes are in a hotspot region. The obtained probabilities 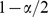 and 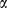 must correspond to the alternating pattern in Figure 7. Therefore, the order is again 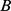, 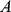, 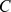 or 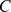, 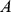, 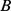, with 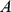 situated at equal distances from 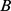 and 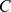. But are the probabilities consistent? In the jumping model, one could easily show that 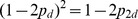. Therefore, we must verify that 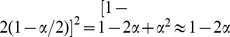, so we will need 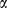 to be small enough. Note also that if 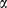 is small enough, the probability that 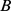 and 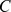 recombine is 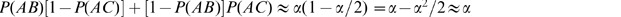, which is consistent. Moreover, the probability of a double crossover is 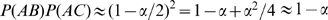, which is the proportion of offsprings in Table 5 that exhibit the double crossover.

## A Possible Delivery Method

Here's a possible method for delivering the content of this exposition to students:

1. Describe the recombination process and genetic linkage with the uniform 1-crossover model as a hypothetical prototype, and explain how genetic mapping can be done based on observed probabilities. Introduce hotspots as an exception to the normal behavior of recombination.
2. As part of a homework assignment, ask which biological properties are satisfied by the uniform 1-crossover model and which are not. Assume that 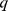 in the “Segregation” section is 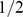. In addition, ask the students to solve a genetic mapping problem with the biological properties in mind and determine whether hotspots are involved or not.
3. (optional) As an advanced question, ask to prove that a uniform 2-crossover model breaks the linkage property.
4. Provide solutions and briefly go over them in class. Introduce Markov chains and the jumping model.
5. As a programming assignment, ask to simulate the jumping model with various values of the parameter 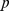 and observe how the probability of recombination changes with distance. Assume that 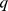 in the “Segregation” section is 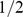.
6. Provide solutions and wrap up by explaining some of the properties of a Markov chain through the jumping model, including the ability to model hotspots.

## Uniform 2-Crossover Model

The derivation of the result is as follows:
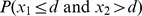


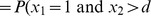


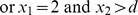


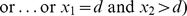
By the exclusivity of events, this is
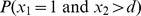


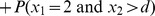


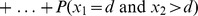


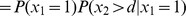


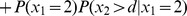


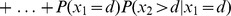
and since 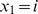 means 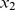 is in 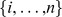, this is
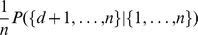


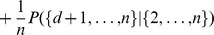


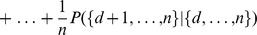


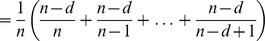


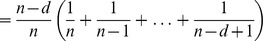


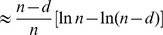
when 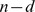 is large.

## Markov Transitional Probabilities

The proof is by induction where 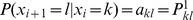 is the base case.
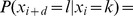


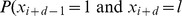


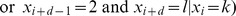
By exclusivity of the two events, this is:
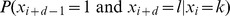


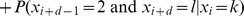
Note that
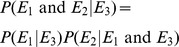
which can be derived from the definition of conditional probability. Therefore, we can rewrite the above as:
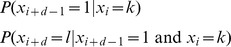


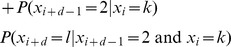
By the Markov property this is:
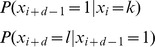


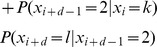


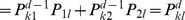
The equality before last represents the inductive step of the proof. The last equality follows immediately from the definition of matrix multiplication.

## Recurrence for 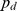


Knowing that 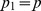, we have a recurrence for 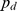 that we can solve, 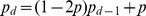. To obtain 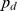 we multiply 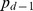 by 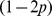 and add 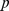. Here are a few attempts:
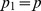


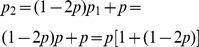


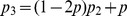


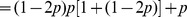


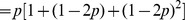


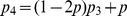


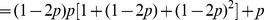


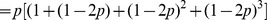
We can easily generalize those attempts to obtain a geometric series:
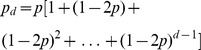


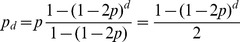



## Conclusion

I am not aware of any other exposition of chromosomal crossover, recombination, genetic linkage, hotspots, and genetic mapping that takes the approach outlined herein. The approach represents a simple and modern treatment of an ancient subject, without a compromise of its scientific and mathematical integrity.

The reader should find an insightful explanation with a focus on reinforcing the ideas by exposing them in different settings. In addition, there is an attempt to introduce the reader to the process of modeling by showing what works and what doesn't. Most importantly, this should provide an early chance to convey to our students that biology is a computational science.

## Disclaimer

I ignored some of the biological detail in favor of simplicity and consistency. Keep in mind, however, that in biology there is always an exception to the rule!

## Further Readings

There is no explicit referencing in the text. This is intentional. I used what everyone would now consider folklore from biology, probability, and calculus. All can be found in textbooks, even elementary ones. For the interested reader, however, and in addition to any introductory texts on probability and calculus, here is a list (in alphabetical order by author) of book chapters that will provide enough background for further endeavors.

1. Gallager RG (1996) Finite State Markov Chains. In: Discrete Stochastic Processes (pp. 103–112). Norwell, MA: Kluwer Academic Publishers.
2. Hunter LE (2009) Evolution. In: The Process of Life: An Introduction to Molecular Biology (pp. 19–47). Cambridge, MA: The MIT Press.
3. Lovász L, Pelikán J, Vesztergombi K (2003) Combinatorial Probability. In: Discrete Mathematics: Elementary and Beyond (pp. 77–80, Uniform Probability). New York, NY: Springer.
4. Stein C, Drysdale RL, Bogart K (2011) Probability. In: Discrete Mathematics for Computer Scientists (pp. 276–279, Conditional Probability). Boston, MA: Pearson Education Inc. (Addison-Wesley).
5. Pevzner PA (2001) Computational Gene Hunting. In: Computational Molecular Biology: An Algorithmic Approach (pp. 1–18). Cambridge, MA: The MIT Press.

